# 2299: Targeted eccentric motor control to improve locomotion after incomplete spinal cord injury

**DOI:** 10.1017/cts.2017.220

**Published:** 2018-05-10

**Authors:** Kevin O’Brien, Michele Basso, James Schmiedeler

## Abstract

OBJECTIVES/SPECIFIC AIMS: Incomplete spinal cord injury (iSCI) is a life-long disability that typically results in a profound loss of locomotion capability. Current rehabilitation methods rarely restore full community ambulation, which in turn limits quality of life. Most individuals with iSCI exhibit persistent deficits in eccentric muscle control and reach recovery plateaus below the levels necessary for independent community ambulation. Eccentric motor control is essential during the weight acceptance phase of gait, which is emphasized during downhill walking. METHODS/STUDY POPULATION: The overground locomotion of subjects with chronic iSCI was analyzed both prior to and following a 12-week downhill body-weight-supported treadmill training regimen and compared to that of matched healthy controls in terms of kinematics, kinetics, and EMG activation. RESULTS/ANTICIPATED RESULTS: We expect to find significant differences between the controls and subjects with iSCI, with deficits in eccentric motor control accounting for some of these differences. In addition, we expect the downhill training to yield significant improvement in eccentric muscle control that translates into improvements in functional, overground walking for the subjects with iSCI. DISCUSSION/SIGNIFICANCE OF IMPACT: The goal is to determine if downhill training can improve eccentric motor control and extend recovery beyond established plateaus. OpenSim modeling of the experimental data will help quantify changes in eccentric control of individual muscles to clarify where specific gains are made.

